# Confocal Microscopy of Unfixed Breast Needle Core Biopsies: A Comparison to Fixed and Stained Sections

**DOI:** 10.1186/1471-2407-9-265

**Published:** 2009-08-03

**Authors:** Linda M Schiffhauer, J Neil Boger, Thomas A Bonfiglio, James M Zavislan, Margarita Zuley, Christi Alessi Fox

**Affiliations:** 1 Department of Pathology and Laboratory Medicine, University of Rochester Medical Center, 601 Elmwood Avenue, Box 626 Rochester, NY 14642, USA; 2 Lucid, Inc., 2320 Brighton Henrietta Town Line Road, Rochester, NY 14623, USA; 3 Department of Pathology and Laboratory Medicine, Rochester General Hospital, 1425 Portland Avenue, Rochester, NY, 14621, USA; 4 University of Rochester, The Institute of Optics, Wilmot 317, Rochester, NY 14642, USA; 5 The Elizabeth Wende Breast Clinic, 170 Sawgrass Drive, Rochester, NY, 14620, USA; 6 University of Rochester Medical Center, 601 Elmwood Avenue, Box 626 Rochester, NY 14642, USA; 7 Magee-Women's Hospital of UPMC, 300 Halket Street, Pittsburgh, PA 15213, USA

## Abstract

**Background:**

Needle core biopsy, often in conjunction with ultrasonic or stereotactic guided techniques, is frequently used to diagnose breast carcinoma in women. Confocal scanning laser microscopy (CSLM) is a technology that provides real-time digital images of tissues with cellular resolution. This paper reports the progress in developing techniques to rapidly screen needle core breast biopsy and surgical specimens at the point of care. CSLM requires minimal tissue processing and has the potential to reduce the time from excision to diagnosis. Following imaging, specimens can still be submitted for standard histopathological preparation.

**Methods:**

Needle core breast specimens from 49 patients were imaged at the time of biopsy. These lesions had been characterized under the Breast Imaging Reporting And Data System (BI-RADS) as category 3, 4 or 5. The core biopsies were imaged with the CSLM before fixation. Samples were treated with 5% citric acid and glycerin USP to enhance nuclear visibility in the reflectance confocal images. Immediately following imaging, the specimens were fixed in buffered formalin and submitted for histological processing and pathological diagnosis. CSLM images were then compared to the standard histology.

**Results:**

The pathologic diagnoses by standard histology were 7 invasive ductal carcinomas, 2 invasive lobular carcinomas, 3 ductal carcinomas in-situ (CIS), 21 fibrocystic changes/proliferative conditions, 9 fibroadenomas, and 5 other/benign; two were excluded due to imaging difficulties. Morphologic and cellular features of benign and cancerous lesions were identified in the confocal images and were comparable to standard histologic sections of the same tissue.

**Conclusion:**

CSLM is a technique with the potential to screen needle core biopsy specimens in real-time. The confocal images contained sufficient information to identify stromal reactions such as fibrosis and cellular proliferations such as intra-ductal and infiltrating carcinoma, and were comparable to standard histologic sections of the same tissue. Morphologic and cellular features of benign and cancerous lesions were identified in the confocal images. Additional studies are needed to 1.) establish correlation of the confocal and traditional histologic images for the various diseases of the breast; 2.) validate diagnostic use of CSLM and; 3.) further define features of borderline lesions such as well-differentiated ductal CIS vs. atypical hyperplasia.

## Background

The "Gold Standard" for diagnosis of breast disease is tissue biopsy followed by histological processing and pathologic diagnosis. A frequently used method to perform a breast biopsy is needle core biopsy, which is less invasive than surgical biopsy and allows tissue to be removed from the breast with minimal scarring and no deformity of the breast. The procedure generally produces a specimen 1–3 mm in diameter and 0.3–2 cm long depending on the nature of the lesion. The specimens are fixed in buffered formalin and submitted for histological processing and evaluation. Specimen adequacy can be assessed in a limited fashion by radiographing the specimen core biopsies to verify removal of calcifications, if calcifications were the finding being targeted for sampling. However, specimen adequacy cannot accurately be confirmed at the time of biopsy and must await histologic examination. Further, if there are no calcifications in the targeted area, no specimen imaging is routinely done at the time of core biopsy. Potentially, this delay in determining specimen adequacy could be mitigated with the use of confocal scanning laser microscopy (CSLM). In addition, CSLM could provide the clinician with a preliminary screening diagnosis within minutes and permit immediate re-biopsy at the point of care, if necessary.

Reflectance CSLM is an optical imaging technique that provides digital images of nuclear and cellular morphology of tissue [1]. It utilizes optical sectioning to form an image of a single horizontal plane either on the surface of the sample or within the sample to a depth of 300 μm. The image records the light backscattered from intra- and inter-cellular constituents within the tissue. The technology has been used non-invasively in human skin in-vivo, producing 3–5 μm thick horizontal sections and a lateral resolution of 0.5–1.0 μm, which compares favorably with the typical 5 μm thick sections prepared for conventional histology. The technique has been applied intra-operatively for the assessment of parathyroid glands, as an ex-vivo application in several studies involving Mohs micrographic surgery and the use of contrast agents, and in pilot studies of ex-vivo hepatic and pancreatic tissues [2-8]. Reflectance confocal imaging has also been applied to imaging of breast cancer in mouse and human models [9-11].

Confocal imaging of breast needle core biopsies for pathological examination prior to standard histopathology can be accomplished with minimal processing of the tissue and no tissue damage in a fashion identical to published reports for CSLM of ex-vivo skin specimens [12]. Because the images represent the spacial variation of optical backscatter within the tissue, contrast agents such as acetic acid or citric acid can be used to enhance the optical contrast of the nucleus relative to the extra-cellular structures such as collagen [13]. In this study, we used CSLM to image breast needle core biopsy specimens, representing a range of breast conditions, during a patient's visit at a radiology practice specializing in breast care, and created a representative set of confocal images with comparative histology.

## Methods

Forty-nine patients underwent needle core biopsy between August 2001 and January 2002 at a large, free-standing breast diagnostic clinic (Elizabeth Wende Breast Clinic, Rochester, NY). This study has been reviewed and approved by an Institutional Review Board (IRB) at the University of Rochester Medical Center, Rochester, NY. The confocal imaging was performed at the clinic at the time of biopsy. Based on radiologist examination, the lesions were characterized using the Breast Imaging Reporting And Data System (BI-RADS). Biopsied cases were assigned as either category 4 or 5. Lesions in category 4 are defined as a suspicious abnormality, and lesions in category 5 as highly suggestive of malignancy. One category 3 case was also biopsied and submitted for imaging during this study. Category 3 is defined in BI-RADS as probably a benign finding.

The confocal microscope used in this study was the commercially available VivaScope 2000 (Lucid, Inc., Rochester, NY), designed specifically for imaging freshly excised surgical tissues. Details of the optical design of the confocal microscope used in this study have been published previously [14]. The system includes a diode laser illumination at a wavelength of 830 nm, maximum illumination power of 30 mW and a 30×, 0.9 numerical aperture water immersion objective lens. The lateral resolution with the 30× objective lens was 1 μm, and the vertical (axial) resolution was < 5 μm. The maximum depth of imaging was approximately 300 μm.

Core biopsies were acquired using either a vacuum-assisted or non vacuum-assisted biopsy devices (11-gauge Mammotome, Ethicon Endosurgery, NJ, 14-gauge Tru-Cut, Baxter Healthcare, CA, 14-gauge Bard, AZ). The gauge of the needle was selected by the radiologist. The freshly excised needle core specimen contained 2–7 (an average of 4.1) pieces of tissue. They were radiographed to identify the pieces that contained calcium, and the piece with the highest density of calcium was selected for imaging. If the specimen did not contain calcium, the piece with the least amount of fat was selected for imaging. The remaining pieces were immediately fixed in formalin. Specimens were excluded from the study if they contained only one piece of tissue so that there would be no potential risk to standard histological processing and subsequent pathological diagnosis. Samples used in this study were fixed in formalin within 20 minutes of removal.

In this study we used two contrast agents, a 5% citric acid solution and glycerin USP. Samples treated with these contrast agents show enhanced nuclear detail and reduced stromal reflectance. The 5% citric acid affects the nuclear reflectance in the same way as 5% acetic acid does, causing compaction of the chromatin, increasing the backscattered light and making the nuclei appear bright in confocal images. The refractive index of glycerin matches that of collagen, reducing its reflectance. Non-adipose stromal areas in the core biopsy specimens treated with glycerin typically became completely translucent and facilitated the ability to image into the specimen.

Core specimens were submerged in a 5 ml fresh solution of 5% citric acid by mixing 2.5 ml of 10% citric acid and 2.5 ml of 0.9% sodium chloride irrigation solution (saline) for 30 seconds. The specimen was then removed from the solution, placed with a drop of glycerin in a tissue cassette and encapsulated with a second adhesive window that stabilized the tissue and prevented drying. The cassette was photographed and placed on the tissue stage of the microscope. The tissue was brought into focus, the laser power was adjusted for optimal image quality, and a composite mapping sequence was initiated. The composite map is a series of full field (475 × 475 μm) images in a single, horizontal plane that are automatically captured and "stitched" together to form a composite image that ranged from 4 × 4 mm to 12 × 12 mm square. This mosaic of images "cuts" an optical section into the specimen close to its surface. Areas of interest were identified and real-time imaging was performed in a 475 × 475 μm field. In areas of interest, a stack of 16 images was captured within the tissue, with each sequential image corresponding to a 3.175 μm step deeper into the tissue. The thickness of a single 16-image stack was approximately 50.4 μm. Sequential stacks of images were captured as long as image quality and resolution were maintained, to a maximum depth of 300 μm. Individual full field images were captured as needed. The complete imaging process took 10–20 minutes, although initial images were obtained in less than five minutes. Following imaging the tissue piece was fixed in buffered formalin along with the rest of the specimen and submitted for standard histological processing (Rochester General Hospital). The unaltered confocal images were reviewed and comparable photomicrographs of the histology were taken.

To evaluate the influences of the contrast agents on immunohistochemical staining, we undertook an IRB-approved (University of Rochester Medical Center) compatibility study on waste mastectomy tissue to examine the effect of different combinations of 10% citric acid, 10% buffered citric acid, 5% acetic acid, glycerin USP, and a saline bath on ER/PR and HER-2/nu stains. The intent of the study was to test the effects of citric acid and acetic acid at the same molarity on breast tissue. The citric acid was buffered with 0.803 M sodium citrate to a pH of 2.4, to match the pH of 5% acetic acid and closely match its molarity of 0.839 M. We compared the appearance of immunohistochemical stains on specimens that were treated with contrast agents to control specimens that were untreated. Following treatment, all specimens were fixed in buffered formalin, submitted for standard histologic processing (University of Rochester Medical Center), and submitted for ER/PR staining, and HER-2/nu staining if cancer was suspected. Pathologists reviewed the sections and stained tissues for artifacts and immunological staining effects outside that typically seen.

## Results

Needle core biopsy specimens were imaged from 49 patients whose breast lesions had been categorized as BI-RADS 3, 4 or 5. The pathologic diagnoses from standard histology were 7 invasive ductal carcinomas, 2 invasive lobular carcinomas, 3 ductal carcinomas in-situ, 21 fibrocystic changes/proliferative conditions, 9 fibroadenomas, 5 other/benign; two were excluded due to imaging difficulties.

Confocal images comparable to standard pathological sections were taken from the same piece of tissue, but not necessarily of the identical field because of differing tissue orientation and sectioning depth. Samples treated with 5% citric acid solution and glycerin showed enhanced nuclear detail and reduced stromal reflectance. The confocal images taken from the needle core biopsy samples showed a range of morphologic features associated with benign and cancerous lesions when reviewed by the three pathologists. Radiological and H&E data from 7 cases are shown (Table 1). Representative confocal images (A) and corresponding H&E images (B) are shown in the figures [Figures, 1, 2, 3, 4, 5, 6 &7]. All scale bars are 50 μm in length.

**Table 1:** Representative Cases

Radiologic Impression/Technique	Patient	BI-RADS Category	Needle Gauge (G)	Pieces of tissue in specimen	Pieces of tissue imaged	Pathologic diagnosis
Calcium positive/mammogram	1	4	11	7	1	Fibrocystic changes with calcifications
mass/mammogram, ultrasound	2	4	14	4	1	Fibrocystic changes with calcifications
mass/sonogram	3	3	14	3	1	Fibrocystic changes with apocrine metaplasia
Calcium positive/mammogram	4	4	11	5	1	Ductal carcinoma in situ
Speculated mass/Mammogram, sonogram	5	4	14	3	1	Infiltrating ductal carcinoma
Mass/mammogram, ultrasound	6	5	14	2	1	Infiltrating lobular carcinoma
Palpable mass/ultrasound	7	5	14	5	1	Poorly differentiated infiltrating ductal carcinoma

**Figure 1:**
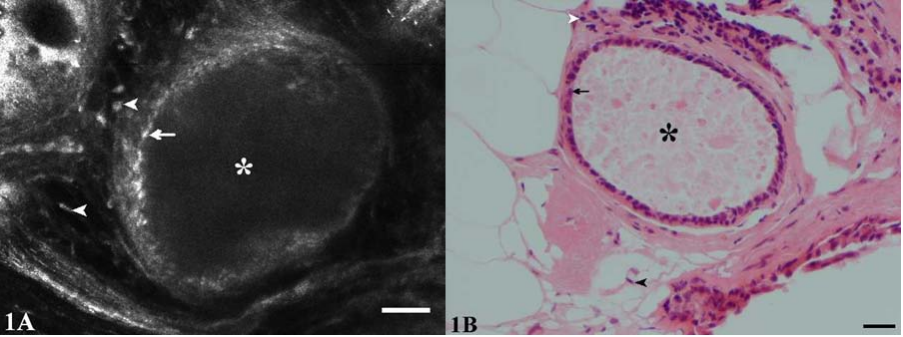
**Fibrocystic changes**. CSLM (A) and H&E (B) images show the lumen of a cyst (*) lined by a layer of small, somewhat flattened epithelial cells (arrows). The nuclei of stromal cells (spindle shaped fibroblasts and rounded lymphocytes) are evident adjacent to the cyst (arrowheads).

**Figure 2:**
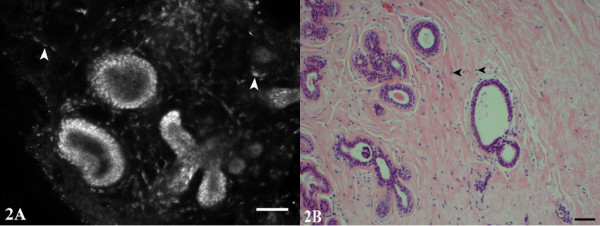
**Fibrocystic changes**. CSLM (A) and H&E (B) images show the microanatomy of the branching terminal duct lobular unit and the epithelial lining of a microcyst. Fibroblasts in the surrounding stroma contain bright nuclei (arrowheads).

**Figure 3:**
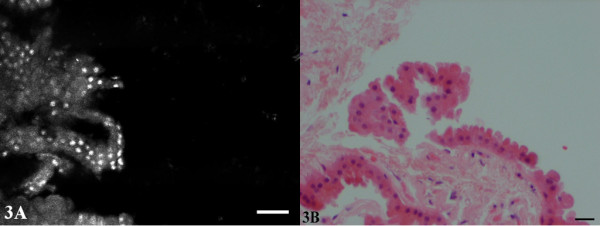
**Apocrine metaplasia**. The intensity of the nuclei in the CSLM image (A) may be attributed to better permeation of the citric acid solution in this partially fragmented specimen. The fine granularity of the cytoplasm corresponds to the eosinophilic granularity of apocrine cells in the H&E (B).

**Figure 4:**
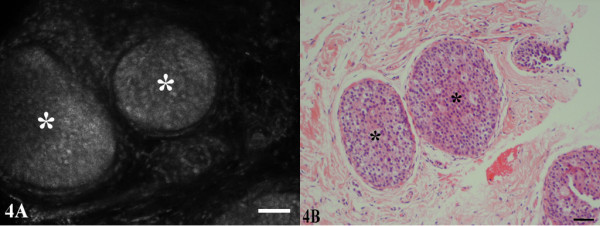
**Ductal carcinoma in situ, nuclear grade 2**. (A) Lumens of the ducts are filled with neoplastic cells with nuclear atypia (*). The nuclei in the CSLM images, represented by the irregular oval to circular granular white structures, are crowded and abnormally arranged. (B) The distinction between atypical hyperplasia and low grade ductal carcinoma in situ cannot always be made on the basis of the CSLM image.

**Figure 5:**
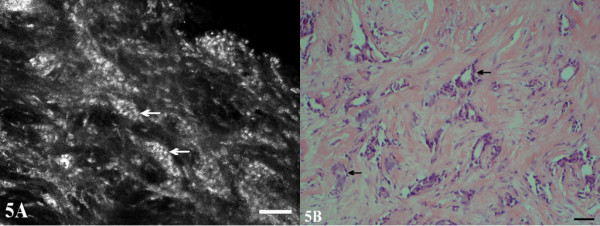
**Infiltrating ductal carcinoma, nuclear grade 1 with tubular features**. (A) The well-defined nature of the infiltrating groups of cells and the relatively small, uniform nuclei (arrows) is evident in both images. Sequentially captured 16-image stacks demonstrated central lumens in some of the groups. The lumens of the tubular-like structures are better defined in the H&E image (B).

**Figure 6:**
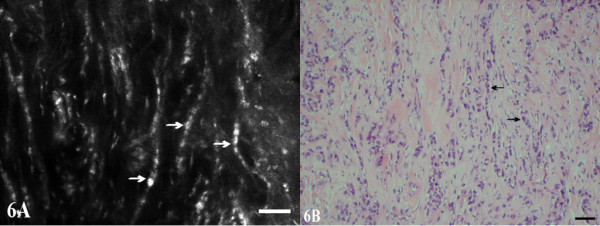
**Infiltrating lobular carcinoma, nuclear grade 2**. Small malignant cells are infiltrating the breast tissue in single file arrangements (arrows) and small nests in images A and B. Nuclear pleomorphism is shown.

**Figure 7:**
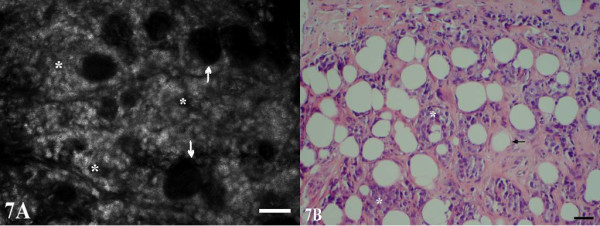
**Poorly differentiated infiltrating ductal carcinoma, nuclear grade 3**. Although the margins of the nuclei are not sharply defined in CSLM (A), the arrangement of tumor cells in diffusely infiltrating nests and aggregates (*) is evident. Residual fat cells are seen as dark, round spaces (arrows). The H&E image (B) shows sharper definition of the nuclear features in this case.

The results of the compatibility study (Table 2) indicated that in samples of specimen 7, which was ER positive, a solution with a pH ≤ 2.4 induced a false negative ER stain (Figure 7B, C). In specimen 1, which was PR control positive, a solution with a pH < 2.4 induced a false negative PR stain. Glycerin USP was not found to have any effect on either ER/PR or HER-2/nu staining. None of the agents induced a false positive or negative HER-2/nu stain. Hematoxylin and eosin staining was not affected by any of the preparations.

**Table 2:** Compatibility Study Results

Sample†	Preparation* Protocol	ER		PR		HER 2/nu		Pathologic diagnosis
		Normal	Cancer	Normal	Cancer	Normal	Cancer	
1A	1	NA	+	NA	+	NA	-	DCIS
1B	2	NA	+	NA	-	NA	-	DCIS
1C	3	NA	+	NA	+	NA	-	DCIS
2A	1	NA	-	NA	-	NA	+	DCIS
2B	4	NA	-	NA	-	NA	+	DCIS
2C	5	NA	-	NA	-	NA	+	DCIS
3A	1	+	NA	+	NA	NA	NA	Normal
3B	6	+	NA	+	NA	NA	NA	Normal
3C	5	+	NA	+	NA	NA	NA	Normal
5A	1	NA	-	NA	-	NA	+	DCIS
5B	5	NA	-	NA	-	NA	+	DCIS
5C	4	NA	-	NA	-	NA	+	DCIS
6A	1	+	-	+	-	NA	-	Invasive DC
6B	4	NA	-	NA	-	NA	-	Invasive DC
6C	5	NA	-	NA	-	NA	-	Invasive DC
6D	8	NA	-	NA	-	NA	-	Invasive DC
7A	1	+	NA	+	NA	NA	NA	Normal
7B	9	-	NA	+	NA	NA	NA	Normal
7C	10	-	NA	+	NA	NA	NA	Normal
7D	5	+	NA	+	NA	NA	NA	Normal
8A	5	+ weak	NA	+	NA	NA	NA	Normal (atrophic)
8B	1	+ weak	NA	+	NA	NA	NA	Normal (atrophic)
8C	1	+ weak	NA	+	NA	NA	NA	Normal (atrophic)
9A	5	NA	-	NA	-	NA	-	Invasive DC
9B	1	NA	-	NA	-	NA	-	Invasive DC
9C	1	NA	-	NA	-	NA	-	Invasive DC
10A	5	+	NA	+	NA	NA	NA	Normal
10B	3	+	NA	+	NA	NA	NA	Normal
10C	11	+	-	+	-	NA	-	Normal and DCIS
10D	1	+	-	+	-	NA	-	Normal and DCIS

†While 10 cases were studied, sample 4 was excluded due to inadequate tissue handling. * Preparation Protocol: Preparation 1: Control; Preparation 2: Unbuffered 10% citric acid (1 min) and saline bath (5 min); Preparation 3: Buffered 10% citric acid (1 min) and glycerin (3 min) without saline; Preparation 4: Glycerin (3 min) without saline; Preparation 5: Buffered 10% citric acid (1 min) and glycerin (3 min) and saline bath; Preparation 6: Buffered 10% citric acid (1 min) without saline bath; Preparation 7: 5% Acetic acid (5 min) without saline bath; Preparation 8: Glycerin (3 min) with saline bath (5 min); Preparation 9: Unbuffered 10% citric acid (1 min) and glycerin (3 min) with saline bath (5 min); Preparation 10: 5% Acetic acid (1 min) and glycerin (3 min) without saline bath; Preparation 11: Mixture of buffered 10% citric acid and 5% acetic acid (1 min) and glycerin (3 min) without saline bath.

## Discussion

The results of the breast needle core study confirm that confocal images can be safely obtained without compromising the ability to make a diagnosis by standard histopathologic preparation. The compatibility study indicated that current immunohistochemical stains are not affected by citric acid or acetic acid if the pH of the contrast agents is buffered to ph>2.4 and the samples are bathed in saline prior to fixation. Also, this study illustrates that CSLM can image features important in the differential diagnosis of breast disease from a range of patients that are presenting for a percutaneous needle core biopsy. However, this study was not structured to determine the sensitivity and specificity of confocal diagnostic criteria. The three pathologists that reviewed the confocal images were aware of the diagnoses from the histologically prepared specimens and were able to view the case slides while viewing the images. Overall, when viewing the breast tissue using confocal imaging, the architectural and cytologic features characteristic of each diagnosis were analogous to those seen using standard histologic processing. The diagnosis most easily made was distinguishing invasive carcinoma from benign tissue. The architectural patterns of invasion characteristic of both invasive ductal and lobular carcinoma were easily seen. Fibrosis was distinguished without difficulty from invasive carcinoma by cellularity, morphology of the nuclei, and the relationship of the nuclei to each other. For normal breast tissue, the architecture of ducts, terminal ducts and acini was clearly visible and benign alterations of these structures such as cysts, apocrine metaplasia and fibroadenomas were obvious. Intraductal proliferations, such as usual ductal hyperplasia and ductal carcinoma in situ, showed architectural and cytologic features in confocal imaging equivalent to those seen using standard histology. Limitations that are typically encountered in standard histology, such as distinguishing atypical proliferations and low grade ductal carcinoma in situ, are expected in confocal imaging as well. One challenge for confocal imaging is viewing specimens composed predominantly of fatty tissue; however, most clinically significant lesions are not expected to be associated primarily with fat.

There are three principal technical challenges in imaging needle-core breast specimens with CSLM. The first is the limited sampling of the tissue. The second is the heterogeneous nature of breast tissue. The third is limited reflectance contrast of the epithelial tissue.

### Sampling

The volume of tissue imaged precluded the ability to completely screen the specimen. Only one mosaic was taken, and from this mosaic multiple image stacks of suspicious regions were acquired. This was due to limitations in the imaging speed of the system and the protocol requirements to fix the tissue within 20 minutes. Because of the limited sampling, frank cancers were often easily seen, but localized ductal carcinoma in situ could be overlooked. Sampling can be improved through enhancements in hardware and software. In this study the mosaic was taken over a square region that circumscribed the tissue, but much of the imaged area did not contain tissue. New versions of the software application allow for the collection of a 10 × 10 mm color macroscopic photo. From this macroscopic photo, the operation can direct the collection of imaged mosaics and stacks. Also, the tissue cassette that held the specimens did not orient the specimen in a particular direction. Using a specifically designed carrier that would orient the specimens lengthwise, the imaging time could be reduced by aligning the tissue with the mechanical motion. The system used in the study imaged 0.475 × 0.475 mm at 9 frames per second, or 2.0 mm^2 ^per second. A core specimen 1.5 mm in diameter and 10 mm in length aligned in a carrier could be imaged at four depths with a fully optimized system in less than two minutes.

### Heterogeneous nature of breast tissue

The depth of imaging (less than 0.3 mm) of the confocal microscope is a limitation for large specimens in that it allows for examination near the surface only. This depth limitation is related to refractive index inhomogeneity of the tissue and the large numerical aperture (0.9) of the imaging system. Of the three main types of tissue in the breast, the adipose tissue limits imaging depth the most. The refractive index of human mesenteric adipose tissue is 1.467 versus 1.41 for the surrounding collagen [15]. This refractive difference induces large optical abberations when attempting to image through a single layer of adipose tissue. Imaging of epithelium in collagen is possible since, as noted earlier, collagen regions of the core biopsy were translucent after treatment with glycerin due to the index matching of glycerin. The index matching reduces both scattering and image abberations. Ducts and glands could be seen visually as pale white structures inside the translucent specimens. The visibility of epithelial structures is believed to be from enhanced backscatter/aceto-whitening of the cellular tissues. Thus, epithelial regions appeared as bright areas in the mosaic, whereas, the glycerin-perfused fibrous tissue appeared dark. In areas away from adipose tissue, epithelium could be imaged to depths greater than 0.3 mm albeit with less resolution than when at the surface. Epithelial proliferations could be detected but not graded at that depth. With advances in the design of the tissue carrier that would allow for imaging on both sides of the carrier, we believe that it will be possible to image epithelium of smaller pieces of tissue to a combined depth of almost 1 mm.

### Contrast in reflectance confocal imaging

Acetic and citric acid increases the endogenous backscatter from the nucleus to increase the contrast of the images. Increasing the contrast was straight forward with the needle core specimens; a 30 second immersion induced nuclear brightening throughout the imaging depth, although the nuclear brightness was greater near the surface than deep in the tissue. This difference can be attributed to reduced optical resolution deep in the tissue and reduced effect of the acid due to pH buffering of the tissue.

The cancerous regions often appeared brighter relative to the normal epithelium in the surrounding tissue, most likely due to the larger nuclei. However, the citric acid treatment did not provide specific contrast associated with cancer. Recent work with exogenous fluorescent dyes and nano particles that attach to dysplastic or cancerous tissue may provide enhanced cancer specific contrast [16-18]. These dyes and nano particles require more elaborate staining protocols, including additional steps to differentiate the contrast agent. Compatibility studies will need to be carried out to ensure that they do not affect the routine prognostic studies, such as immunohistochemistry and FISH, used in the clinical care of breast cancer patients. However, these agents demonstrate our ability to specifically label cancerous regions and may aid in the rapid detection of cancer in breast specimens in the future.

Confocal scanning laser microscopy has many potential applications in the practice of surgical pathology. Rapid diagnoses or determinations of adequacy could be given on core biopsy specimens, margins could be assessed by either ex vivo tissue imaging or intraoperative consultations at the time of primary surgical excision and CSLM could aid in the selection of sections to submit for microscopic study in large primary excisions or in the evaluation of re-excision specimens. These applications could result in a decreased time to diagnosis, reduced rate of re-excision, lower cost of pathology specimen processing, and improved patient care.

Although the size of this study is limited, the cases imaged represent a spectrum of breast conditions that require biopsy as determined by radiographic examination and patient history. We show that important morphological features of breast conditions can be imaged using CSLM. Our results call for further studies of percutaneously retrieved or open surgical specimens using reflectance or fluorescence confocal imaging with either single wavelength systems or newer multi-wavelength devices. In addition, we advocate the construction of a comprehensive atlas to develop the diagnostic criteria necessary for confocal screen of breast specimens.

## Conclusion

Morphologic and cellular features of benign and cancerous lesions were identified in the confocal images. The images contained sufficient information to identify stromal reactions such as fibrosis, and cellular proliferations such as ductal carcinoma in situ and infiltrating carcinoma, and were comparable to standard histologically-prepared sections of the same tissue. Confocal images can be enhanced by immersing the tissue in 5% citric acid solution for 30 seconds and then immersing and viewing it in glycerin. After confocal imaging, the tissue remains intact and available for standard histological processing, including the use of immunohistological stains. The effects of citric acid and glycerin immersion on subsequent histologic processing are demonstrated. Tissue treated with 5% acetic acid or 5%–10% citric acid should be bathed in saline for 5 minutes prior to fixation, and the pH of either acetic acid or citric acid should be buffered to pH>2.4 to preserve receptor reactivity.

Because reflectance confocal images are visually different from traditionally processed histological sections, an atlas of comparative pathology is needed. To further build an atlas, additional studies are required to develop a comprehensive understanding of the correlation of confocal images to traditionally prepared slides for the various conditions and diseases of the breast.

## Abbreviations

BIRADS: Breast Imaging Reporting And Data System; CSLM: Confocal Scanning Laser Microscopy; ER: Estrogen Receptor; H&E: Hematoxylin and Eosin; HER (HER2/nu): Herceptin; IRB: Institutional Review Board; PR: Progesterone Receptor; FISH: Fluorescent In Situ Hybridization

## Competing interests

LMS declares no financial or non-financial competing interests. JNB declares that he is a shareholder of Lucid, Inc. common stock. TABdeclares no financial or non-financial competing interests. JMZ declares that he was formerly the Vice Present of Technology at Lucid, Inc. At the time this study took place he was managing and currently manages Lucid's intellectual property portfolio on a part-time basis. He is also a shareholder of Lucid, Inc. common stock. MZ declares no financial or non-financial competing interests. CAF is an employee of Lucid, Inc. and a shareholder of common stock in the Company.

## Authors' contributions

LS contributed in the selection, analysis and interpretation of the confocal images and histology as well as drafting of and final approval of this manuscript. JNB contributed in the selection of the seven cases that were represented, the selection of the individual confocal images and histology that were chosen to represent the cases, the drafting of and final approval of the manuscript. TB contributed to the conception, design and execution of the first and second compatibility studies, histological analysis of the specimens in the first and second compatibility studies, the conception and design of the overall study, analysis and interpretation of the confocal images and histology, and the final approval of the manuscript. JZ contributed to the conception, design and execution of the first and second compatibility studies, the conception and design of the overall study, selection of cases that were represented in the study, selection of confocal images presented in the study, drafting of and final approval of the manuscript. MZ contributed in the conception and design of the overall study, selection of specimens core biopsy submitted to the study as well as drafting of the manuscript and final approval. CAF contributed in the design of the study, image acquisition and data management for the study, selection of cases presented in the study, selection of confocal images presented in the study, drafting of the manuscript and final approval of the manuscript. All authors read and approved the final version of the manuscript.

## Pre-publication history

The pre-publication history for this paper can be accessed here:

http://www.biomedcentral.com/1471-2407/9/265/prepub
